# Netrin‐1 upregulates GPX4 and prevents ferroptosis after traumatic brain injury via the UNC5B/Nrf2 signaling pathway

**DOI:** 10.1111/cns.13997

**Published:** 2022-12-05

**Authors:** Yuanda Zhang, Jin Lan, Dongxu Zhao, Cijie Ruan, Jue Zhou, Haoyuan Tan, Yinghui Bao

**Affiliations:** ^1^ Department of Neurosurgery, Renji Hospital, School of Medicine Shanghai Jiao Tong University Shanghai China

**Keywords:** ferroptosis, GPX4, netrin‐1, traumatic brain injury (TBI)

## Abstract

**Aim:**

We aimed to investigate the regulatory role of Netrin‐1 (NTN1) in ferroptosis after traumatic brain injury (TBI) in mice.

**Methods:**

We assessed the expression pattern of NTN1 by RT–PCR, western blot, and immunofluorescence after establishing the TBI model in mice. After treatment with NTN1 shRNA or recombinant NTN1, we determined the biochemical and morphological changes associated with ferroptosis and netrin‐1‐related pathways. We used Nissl staining to assess lesion volume and Morris water maze and beam‐walking test to evaluate ethological manifestation.

**Results:**

The mRNA and protein levels of NTN1 were upregulated after TBI. The application of NTN1 shRNA increased the number of FJB positive cells, malondialdehyde (MDA), and reactive oxygen species (ROSs) levels. However, the application of NTN1 recombinant had the opposite effect. Furthermore, knockdown or inhibition of GPX4, Nrf2, and UNC5B counteracted the effects of NTN1 recombinant. Intravenous injection of NTN1 recombinant reduced neuronal loss after CCI and improved motor and cognitive function.

**Conclusion:**

NTN1 had a neuroprotective effect after TBI and inhibited ferroptosis via activating the UNC5B/Nrf2 pathway. These findings may provide potential therapeutic strategies for TBI.

## Graphical abstract

The schematic diagram of neuroprotective and anti‐ferroptotic effects of Netrin‐1: Upregulation of NTN1 and activation of UNC5B receptor after TBI promote Nrf2 nuclear translocation and enhance GPX4 transcription. Exogenous Netrin‐1 treatment alleviates ferroptosis and improves neurological deficits.

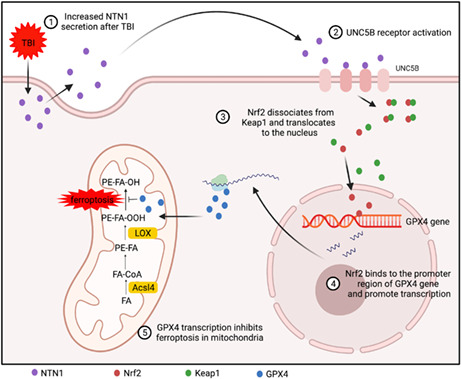

## 1 INTRODUCTION

Traumatic brain injury (TBI) is a severe public health issue worldwide.^1^ The pathophysiology of TBI consists of primary injury and secondary injury. Oxidative stress has been implicated in numerous secondary pathological processes.^2^ As a neuroinflammation‐induced process related to dysfunctional organelles,^3^ oxidative stress generates an end product known as malondialdehyde (MDA). Finally, it activates the signal pathways related to cell death‐like necroptosis,^4^ pyroptosis,^5^ and autophagy.^6^ Therefore, antioxidant therapy serves as an important strategy for the treatment of TBI.^7^

As a recently discovered form of cell death closely related to oxidative stress, ferroptosis was proposed by Dixon in 2012.^8^ There are three main signaling pathways involved in ferroptosis: lipid peroxidation, cystine and cysteine metabolism, and iron accumulation.^9^ As a key enzyme in ferroptotic signaling pathways, glutathione peroxidase‐4 (GPX4) plays an important role in the association of lipid peroxidation and cystine metabolism.^10^ Lipoxygenase (LOX), a key enzyme in the iron‐dependent lipid peroxidation signaling pathways, can be inhibited by GPX4.^11^ In this way, GPX4 protects the cellular membrane and mitochondrial from peroxidation^12^ and therefore rescues cells from ferroptosis. Studies have confirmed that ferroptosis demonstrate a correlation with the occurrence and development of TBI. The increased iron accumulation after TBI leads to lipid peroxidation and ROS production, resulting in the neuronal ferroptosis. Iron chelators can be used to inhibit ferroptosis and alleviate clinical manifestations.^13^ Thus, ferroptosis may be a novel clinical therapeutic direction for TBI.

As one secretory laminin closely related to nerve regeneration,^14^ Netrin‐1 (NTN1) was identified by Tessier–Lavigne in the examination of the dorsal neural tube. Since then, Netrin‐1 has been found to play a critical role in embryonic development like axon guidance, cell migration, morphogenesis, and angiogenesis.^15^ Importantly, later studies found that Netrin‐1 was involved not only in nerve regeneration but also in the pathogenesis of TBI. In a clinical study, Yun Xie found that the expression of Netrin‐1 had an inverse relationship with the severity of TBI.^16^ Noteworthy, the neuroprotective effects of Netrin‐1 were observed to be related to the molecular pathways associated with oxidative stress,^17^, ^18^ and this suggested that Netrin‐1 may be involved in the process of ferroptosis—a form of cell death closely linked to oxidative stress.

In this study, we investigated the mechanism of Netrin‐1 in the process of ferroptosis after TBI. We showed that Netrin‐1 can upregulate the level of Nrf2 through the UNC5B receptor and facilitate its translocation to the nucleus, thereby promoting the transcription of GPX4. The signal transduction can alleviate ferroptosis after TBI and protect the motor and cognitive functions of mice.

## 2 MATERIALS AND METHODS

### 2.1 CCI model

As described,^19^ the moderate CCI model was performed in this study. Briefly, animals were anesthetized with isoflurane (3% for induction and 1.5% for maintenance, oxygen delivered at 0.5 L/min) and mounted and fixed on a stereotaxic device (Stoelting, Italy). A median sagittal incision was performed on the scalp and a hole in the skull (4 mm diameter) was made exposing the dura. The hole was between the left bregma and lambda, whose medial edge 2 mm lateral to the midline. We removed the bone flap and attached a 3.0 mm rounded impacting tip to an electromagnetically controlled impacting device (PinPointTM PCI3000 Precision Cortical ImpactorTM, USA), adjusting the tip vertical to the dura. The machine was set as follows: velocity of 3.0 m/s, deformation depth of 2.0 mm, and dwell time of 180 ms. In the sham group, mice were treated identically except for impacting.

### 2.2 Cell culture and mechanical stretch model

SH‐SY5Y cells were acquired from the Center for Excellence in Molecular Cell Science, Chinese Academy of Sciences (Shanghai, China). Cells were cultured in DMEM (Servicebio, China) supplemented with 10% fetal bovine serum (Absin, China) and 1% penicillin/streptomycin/amphotericin B solution (Beyotime, China), and then exposed to a gas mixture containing 5% CO_2_ and 95% air. Subculturing was carried out every 3 days.

We used the Cell Injury Controller II system (CIC II, Virginia Commonwealth University) to establish the mechanical stretch model. We discarded the supernatants when cell confluence reached 70% and digested the cells with trypsin, making a cell suspension. Cells were seeded at a density of 0.3 × 10^5^/cm^3^ on collagen type I‐precoated 6‐well stress plates (BioFlex, UK). Then, the CIC II system was used to produce a biaxial stretch by 50‐ms burst of nitrogen gas. The adherent cells experienced a 6.5‐mm downward deformation and underwent stretch injury. After 24‐h incubation, cells were applied for the next experiments.

### 2.3 Fluoro‐Jade B (FJB) staining

Fluoro‐Jade B (FJB) staining was used to assess neuronal death. The sections were washed twice with distilled water and immersed in alkaline alcohol solution (1X sodium hydroxide with 50% alcohol) for 5 min. After that, the sections were dehydrated in 70% ethanol and distilled water for 2 min, respectively, and then incubated in 0.06% potassium permanganate solution for 10 min. After rinsing with distilled water, the sections were transferred to 0.0004% Fluoro‐Jade B solution (AG310, Millipore, Germany) for 10 min, and then rinsed in distilled water 3 times. After drying, the slides were coverslipped with DPX nonfluorescent mounting media. The number of FJB+ cells was counted under a microscope (Nikon TE300; Nikon) using NIH Image J software (Bethesda, MD, USA) by a technician who was blind to the experimental design. At least three slides per mouse sample were stained for counting, and four random visual fields from each slide were observed.

### 2.4 Measurement of MDA, ROSs, and glutathione (GSH) levels

Commercially available MDA (S0131, Beyotime, China), ROS (E004‐1‐1, Jiancheng, China), and GSH (S0053, Beyotime, China) kits were used in our study. The operations were carried out according to the instructions of the kit.

### 2.5 Behavioral experiments

Beam‐walking test was used to evaluate the motor function of mice 7 days after CCI modeling. Four weeks after CCI modeling, the learning and memory functions were evaluated using the Morris water maze test.

### 2.6 Statistical analysis

SPSS 26.0 was used for statistical analysis. The Shapiro–Wilk test was used to confirm whether the data fit a normal distribution. Data were summarized as mean ± SD (normally distributed) or median (non‐normally distributed). For normally distributed data, one‐way analysis of variance (ANOVA) was used for multiple group comparisons. The least significant difference (LSD) post hoc test was used to determine statistical differences between groups. Statistical differences between the two groups were compared using Student's *t*‐test. For data that did not conform to a normal distribution, comparisons between groups were made using the Mann–Whitney U test. *p* values less than 0.01 (*p* < 0.01) were considered statistically significant.

## 3 RESULTS

### 3.1 NTN1 expression changes in the injured cortex over time

**FIGURE 1:**
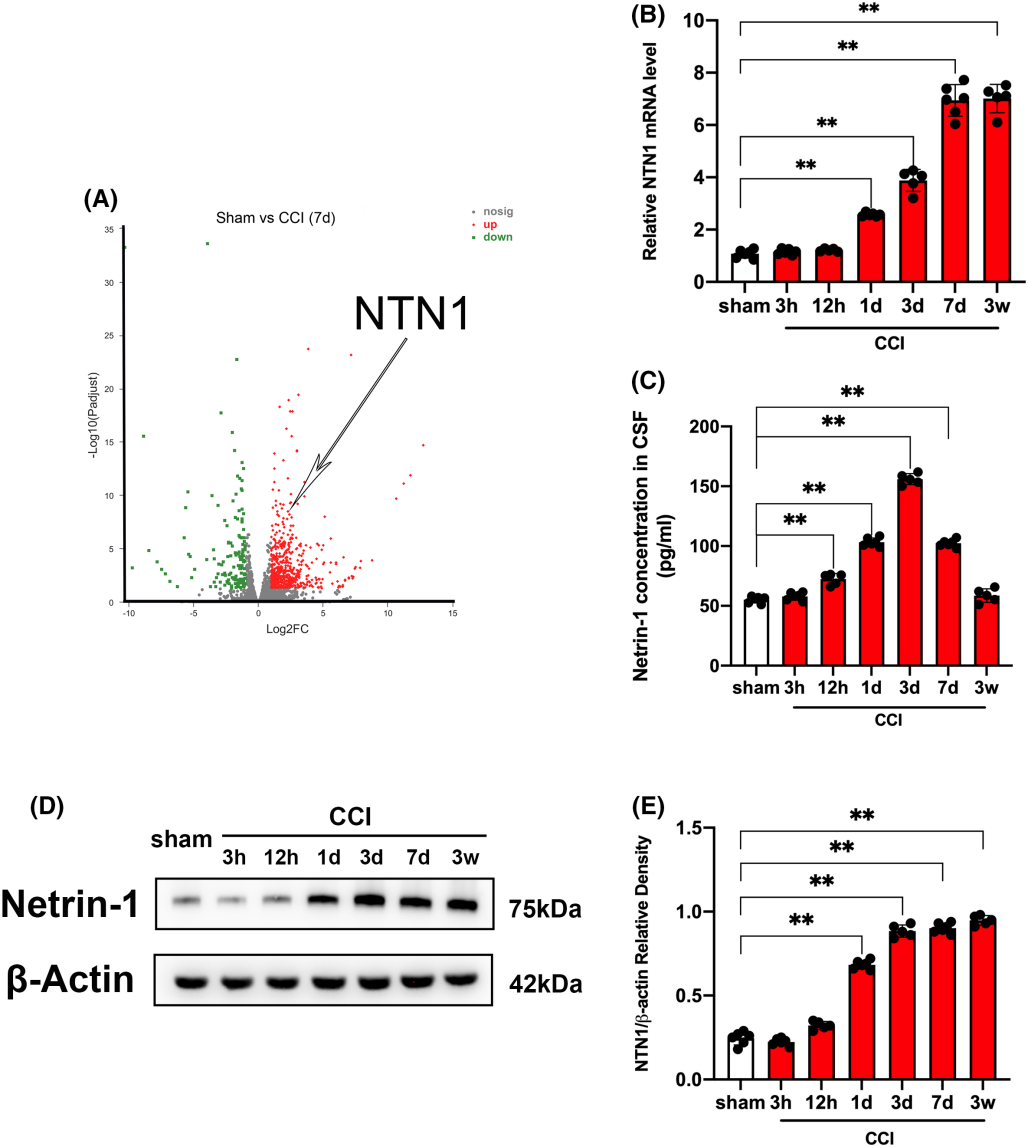
NTN1 expression was upregulated after CCI. (A) Results of RNA sequencing. RNA sequencing was performed on mice injured cortex in sham group (*n* = 3) and CCI group (*n* = 6). Volcano plot shows the differentially expressed genes. Each circle represents a gene. Significantly upregulated genes are highlighted in red and downregulated genes are highlighted in green. (B) NTN1 mRNA content at different time points after CCI. Quantitative real‐time PCR was carried out to determine the NTN1 mRNA content. β‐Actin was used as an internal control and the results were presented as fold change of sham group. (C) Cerebrospinal fluid (CSF) levels of NTN1 measured with ELISA. (D, E) NTN1 protein content determined by western blot at different time points after CCI. β‐Actin was used as control. The data for each group conformed to a normal distribution. Data were analyzed using the one‐way ANOVA with LSD post‐hoc test. **p < 0.01. There was no difference in body weight between mice in each group. *n* = 5 (12 h, 3 days and 3 weeks after CCI, 1 mouse died respectively), *n* = 6 (sham group, 3 h, 1 and 7 days after CCI).

The experimental design is illustrated in Figure S1A. We collected injured cortex 7 days after modeling from 3 mice in sham group and 6 mice in CCI group for transcriptome sequencing. A total of 565 genes were upregulated and 144 genes were downregulated. According to the sequencing results, we found that the expression of NTN1 was highly variable (Figure 1A). In view of the fact that no studies have evaluated the expression of NTN1 at different time points after CCI, we used qPCR and western blotting (WB) to detect the mRNA and protein levels of 3, 12 h, 1, 3, 7 days, and 3 weeks after CCI. Because NTN1 is a secreted protein, we also evaluated its secretion level in the cerebrospinal fluid (CSF). The results of qPCR showed that the mRNA level of NTN1 started to be significantly higher than that of the sham group 1d after CCI, reached the peak at 7 days after CCI, and maintained high expression within 3 weeks after CCI (Figure 1B). The secretion level of NTN1 in CSF was carried out by ELISA kit. The results showed that the concentration of NTN1 in CSF was significantly higher than that in the sham group at 12 h after CCI, reached the peak and began to decline at 3 days after CCI. At 3 weeks after CCI, the concentration of NTN1 in CSF was not significantly different from that in the sham group (Figure 1C). Next, we assessed the expression of NTN1 in the injured cortex by WB and immunofluorescence. The results of WB showed that the protein level of NTN1 was significantly higher than that of the sham group at 1 day after CCI, peaked at 3 days after CCI, and maintained high expression within 3 weeks after CCI (Figure 1D,E). The results of immunofluorescence showed that the fluorescence intensity of NTN1 was significantly higher than that of the sham group at 7 days and 3 weeks after CCI Figure S1B.

### 3.2 Ferroptosis in the injured cortex after CCI

**FIGURE 2:**
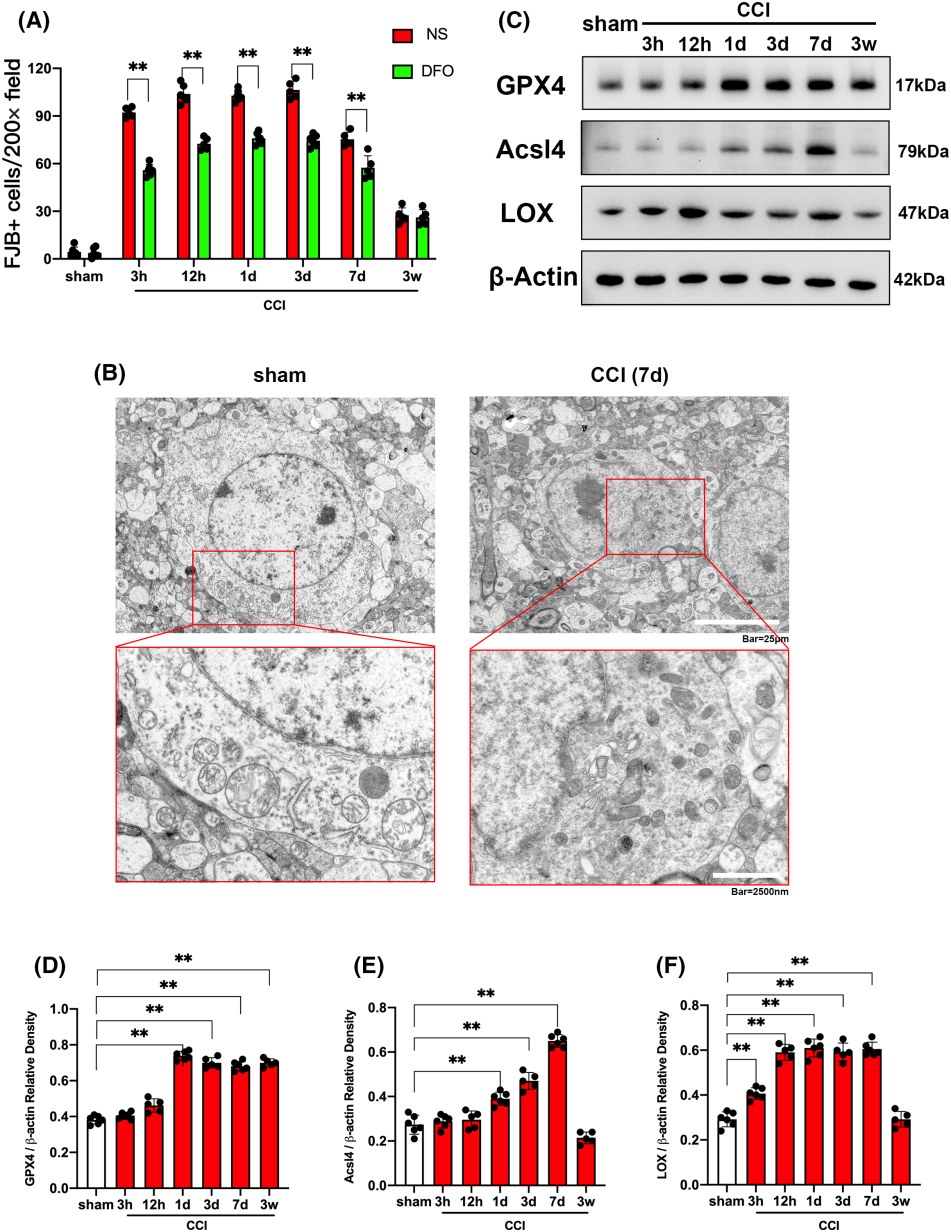
Ferroptosis of injured cortex after CCI. (A) Quantitative analysis of FJB‐positive cells. Data were analyzed using the Mann–Whitney U test. **p < 0.01. There was no difference in body weight between mice in each group. DFO treatment: *n* = 5 (12 h, 7 days and 3 weeks after CCI, 1 mouse died respectively), *n* = 6 (sham group, 3 h, 1 and 3 days after CCI). NS treatment: *n* = 5 (3 h, 3, 7 days and 3 weeks after CCI, 1 mouse died respectively), *n* = 6 (sham group, 12 h and 1 day after CCI). (B) Ultrastructure of the mice injured cortex captured by transmission electron microscopy. Scale bar of upper panel is 25 μm and scale bar of bottom panel is 2500 nm. (C–F) GPX4, Acsl4 and LOX protein content determined by western blot at different time points after CCI. β‐Actin was used as control. The data for each group conformed to a normal distribution. *p* value was determined by ANOVA with LSD post‐hoc test. **p < 0.01. There was no difference in body weight between mice in each group. *n* = 5 (12 h, 3 days and 3 weeks after CCI, 1 mouse died respectively), *n* = 6 (sham group, 3 h, 1 and 7 days after CCI).

We selected 6 mice in CCI+PBS group and 6 mice in CCI+Netrin‐1 group for transcriptome sequencing. The KEGG analysis was performed based on the sequencing results. Our study showed that the application of NTN1 enriched the ferroptosis signaling pathway (Figure S2A). In view of the fact that no studies have confirmed the relationship between NTN1 and ferroptosis, we decided to explore the mechanism of NTN1 in ferroptosis pathways. DFO was applied to chelate ferric ion, and the dead neurons were counted by FJB staining at different time points after CCI. Normal saline (NS) was used as a control. The results of FJB staining showed that at different time points after CCI, the number of FJB positive cells was significantly greater than that in the sham group. DFO treatment could reduce the number of FJB‐positive cells at different time points after CCI (Figures 2A, S2B). Next, we measured the levels of MDA, ROS, and GSH in the injured cortex at different time points after CCI. Comparing the MDA levels of CCI group and sham group, our study found that the MDA level started to be significantly higher than sham group 3 h after CCI, reached a peak 3 days after CCI, maintained a high level of expression within 7 days after CCI, and decreased to the level of sham group 3 weeks after CCI (Figure S2C). Comparing the ROS levels of the CCI group and sham group, we found that the ROS level started to be significantly higher than sham group 3 h after CCI, reached a peak 3 days after CCI, maintained a high level of expression within 7 days after CCI, and decreased 3 weeks after CCI. Notably, ROS levels were significantly higher than those in sham group within 3 weeks after CCI. (Figure S2D). Comparing the GSH levels of CCI group and sham group, we found that the GSH level started to be significantly lower than sham group 3 h after CCI, reached the bottom 3 days after CCI, maintained a low level of expression within 7 days after CCI, and increased 3 weeks after CCI. However, GSH levels were significantly lower than those in the sham group within 3 weeks after CCI (Figure S2E). Furthermore, the results of transmission electron microscopy showed that dense and shrunken mitochondria were highly obvious in the injured cortex 7 days after CCI compared with the sham group (Figure 2B). Next, we assessed the protein expression of GPX4, Acsl4, and LOX, key molecules associated with ferroptosis‐related signal transduction channels, by WB. The results showed that the expression of GPX4 started to be significantly up‐regulated 1 day after CCI, and was significantly higher than that in sham group within 3 weeks after CCI (Figure 2C,D). The expression of Acsl4 started to be significantly up‐regulated 1 day after CCI, and reached a peak 7 days after CCI. The expression of Acsl4 was not significantly different from sham group 3 weeks after CCI (Figure 2C,E). The expression of LOX started to be significantly up‐regulated 3 h after CCI, reached a peak 12 h after CCI, and maintained a significantly high expression within 7 days after CCI. The expression of LOX was not significantly different from sham group 3 weeks after CCI (Figure 2C,F).

### 3.3 NTN1 ameliorated ferroptosis in the injured cortex after CCI

**FIGURE 3:**
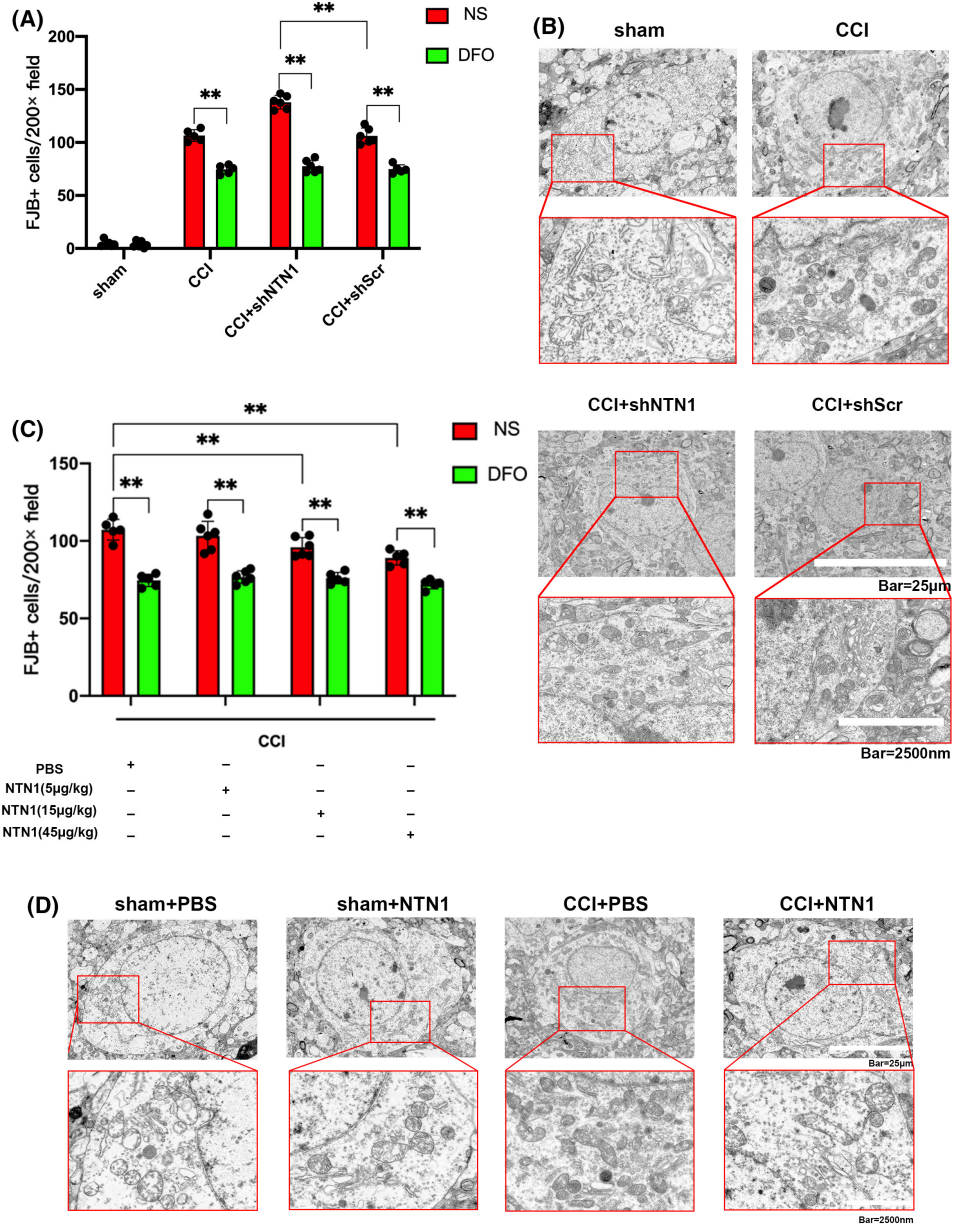
NTN1 ameliorated ferroptosis in the injured cortex after CCI. (A) Quantitative analysis of FJB‐positive cells. The data for each group conformed to a normal distribution. *p* value was determined by ANOVA with LSD post‐hoc test. **p < 0.01. There was no difference in body weight between mice in each group. DFO treatment: *n* = 5 (CCI group and CCI+shScr group, 1 mouse died respectively), *n* = 6 (sham group and CCI+shNTN1 group). NS treatment: *n* = 5 (CCI group, 1 mouse died), *n* = 6 (sham group, CCI+shNTN1 group and CCI+shScr group). (B) Ultrastructure of the mice injured cortex captured by transmission electron microscopy. Scale bar of upper panel is 25 μm and scale bar of bottom panel is 2500 nm. (C) Quantitative analysis of FJB‐positive cells. The data for each group conformed to a normal distribution. *p* value was determined by ANOVA with LSD post‐hoc test. **p < 0.01. There was no difference in body weight between mice in each group. DFO treatment: *n* = 5 (CCI+PBS group, CCI+15 μg/kg NTN1 group and CCI+45 μg/kg NTN1 group, 1 mouse died respectively), *n* = 6 (CCI+5 μg/kg NTN1 group). NS treatment: *n* = 5 (CCI+PBS group and CCI+45 μg/kg NTN1 group, 1 mouse died respectively), *n* = 6 (CCI+5 μg/kg NTN1 group and CCI+15 μg/kg NTN1 group). (D) Ultrastructure of the mice injured cortex captured by transmission electron microscopy. Scale bar of upper panel is 25 μm and scale bar of bottom panel is 2500 nm.

In order to explore the role of NTN1 in ferroptosis after CCI, we applied AAV–NTN1 shRNA to mice (Scrambled shRNA served as a control), and then counted dead neurons by FJB staining. Compared with the CCI + shScr group, the number of FJB positive cells in the CCI+shNTN1 group was significantly increased. After application of DFO, the difference in the number of FJB‐positive cells between the CCI+shScr group and the CCI+shNTN1 group was offset (Figures 3A, S3A). Furthermore, compared with the CCI+shScr group, the levels of MDA (Figure S3B) and ROS (Figure S3C) in the CCI+shNTN1 group were significantly increased, but there was no significant difference in GSH levels between the two groups (Figure S3D). We observed the morphology of mitochondria with transmission electron microscope and found that NTN1 shRNA led to more severe mitochondria shrinkage compared with the control group (Figure 3B).

Next, we applied recombinant NTN1 protein (PBS as control) in the CCI model and reassessed the number of FJB‐positive cells. Compared with the control group, the application of NTN1 recombinant protein at the concentration of 15 and 45 μg/kg significantly reduced the number of FJB‐positive cells. The application of DFO could counteract the effect of NTN1 recombinant protein (Figures 3C, S3E). In addition, the application of NTN1 recombinant protein at the concentration of 5, 15 and 45 μg/kg reduced the level of MDA in brain tissue in CCI model (Figure S3F). Application of NTN1 recombinant protein at the concentration of 15 and 45 μg/kg reduced the level of ROS (Figure S3G) and increased the level of GSH (Figure S3H) in brain tissue. Finally, the transmission electron microscopy showed that the degree of mitochondrial shrinkage was significantly reduced in the CCI+NTN1 group compared with the CCI+PBS group (Figure 3D).

### 3.4 NTN1 inhibited cortical ferroptosis by upregulating GPX4

**FIGURE 4:**
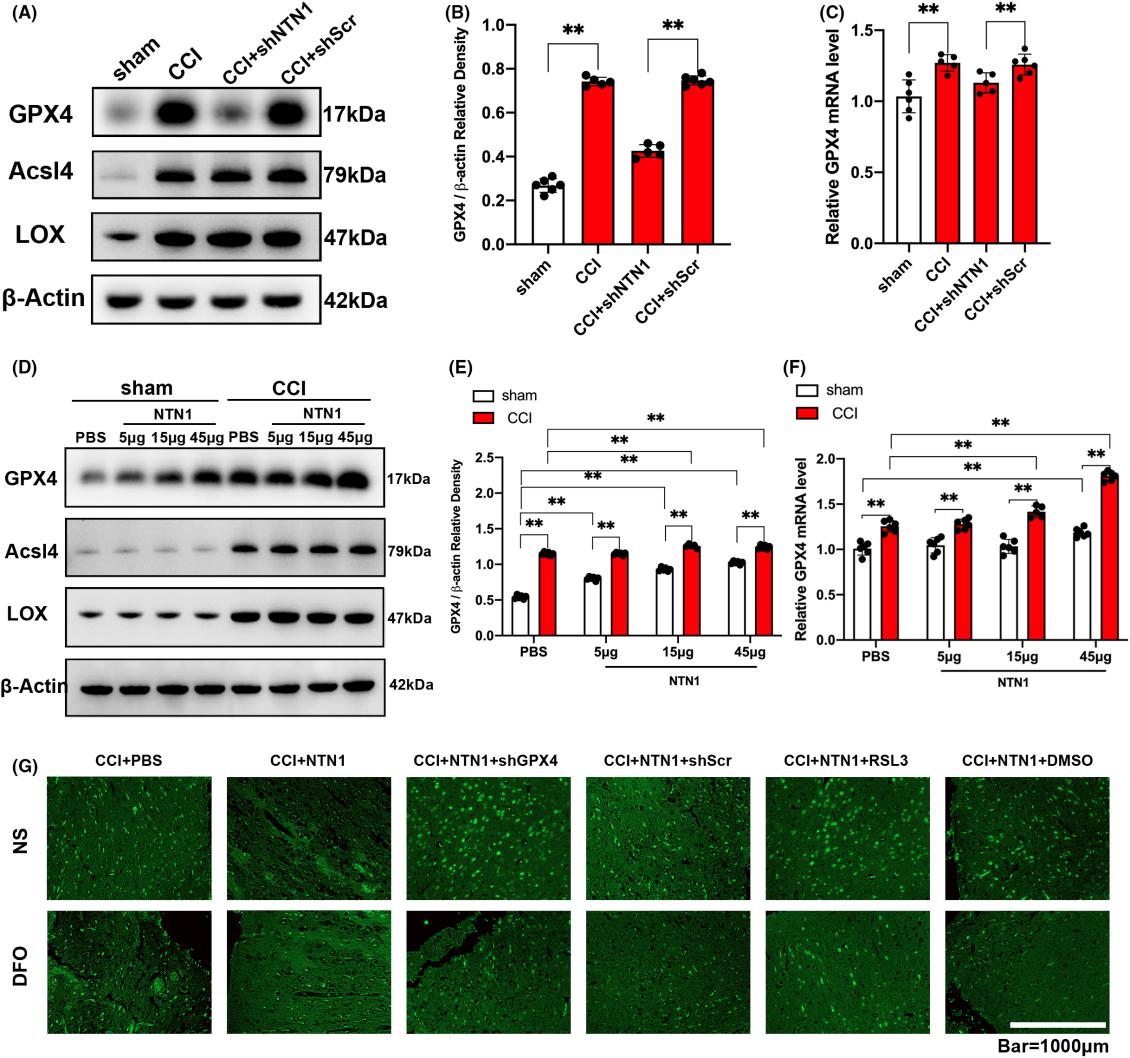
NTN1 ameliorated cortical ferroptosis by upregulating GPX4. (A, B) GPX4, Acsl4 and LOX protein content determined by western blot at 7 days after CCI. β‐Actin was used as control. Histogram shows the quantitative analysis of GPX4 protein content. The data for each group conformed to a normal distribution. *p* value was determined by ANOVA with LSD post‐hoc test. **p < 0.01. There was no difference in body weight between mice in each group. *n* = 5 (CCI group and CCI+shNTN1 group, 1 mouse died respectively), *n* = 6 (sham group and CCI+shScr group). (C) GPX4 mRNA content at 7 days after CCI. Quantitative real‐time PCR was carried out to determine the GPX4 mRNA content. β‐Actin was used as an internal control and the results were presented as fold change of sham group. Data were analyzed using the Mann–Whitney U test. **p < 0.01. There was no difference in body weight between mice in each group. *n* = 5 (CCI group and CCI+shNTN1 group, 1 mouse died respectively), *n* = 6 (sham group and CCI+shScr group). (D, E) GPX4, Acsl4 and LOX protein content determined by western blot at 7 days after CCI. β‐Actin was used as control. Histogram shows the quantitative analysis of GPX4 protein content. Data were analyzed using the Mann–Whitney U test. **p < 0.01. There was no difference in body weight between mice in each group. Sham group: *n* = 6 each. CCI group: *n* = 5 (CCI+15 μg/kg NTN1 group, 1 mouse died), *n* = 6 (CCI+PBS group, CCI+5 μg/kg NTN1 group and CCI+45 μg/kg NTN1 group). (F) GPX4 mRNA content at 7 days after CCI. Quantitative real‐time PCR was carried out to determine the GPX4 mRNA content. β‐Actin was used as an internal control and the results were presented as fold change of sham+PBS group. Data were analyzed using the Mann–Whitney U test. **p < 0.01. There was no difference in body weight between mice in each group. Sham group: *n* = 6 each. CCI group: *n* = 5 (CCI+15 μg/kg NTN1 group, 1 mouse died), *n* = 6 (CCI+PBS group, CCI+5 μg/kg NTN1 group and CCI+45 μg/kg NTN1 group). (G) Representative images of Fluoro‐Jade B (FJB) stained brain sections. Scale bar is 1000 μm.

In order to explore the targets of NTN1 on regulating ferroptosis after CCI, we applied AAV–NTN1 shRNA or NTN1 recombinant protein to mice, and then measured the expression of several key enzymes. After NTN1 shRNA treatment, we assessed NTN1 protein levels by WB and NTN1 mRNA levels by qPCR. We found that NTN1 shRNA treatment significantly downregulated the expression of GPX4 protein compared with the control group, but had no significant effect on the expression of Acsl4 and LOX (Figures 4A,B, S4A,B). At the same time, NTN1 shRNA also reduced the mRNA level of GPX4 (Figure 4C). Furthermore, exogenous NTN1 treatment upregulated the expression of GPX4 in a dose‐dependent manner, but had no significant effect on the expression of Acsl4 and LOX (Figures 4D–F, S4C,D).

Next, we inhibited the expression of GPX4 through AAV–GPX4 shRNA or RSL3, a specific inhibitor of GPX4, to reassess the role of exogenous NTN1 in CCI model. Compared with the control group, GPX4 shRNA or RSL3 treatment partially offset the effect of exogenous NTN1 on reducing MDA (Figure S4E) and ROS (Figure S4F) levels and increasing GSH (Figure S4G) levels in brain tissue after CCI. The FJB staining results showed that when GPX4 was inhibited by GPX4 shRNA or RSL3, application of exogenous NTN1 could not reduce the number of FJB positive cells after CCI. However, DFO treatment offset the effect of GPX4 shRNA or RSL3 (Figure 4G).

### 3.5 NTN1 upregulated GPX4 through UNC5B/Nrf2 pathway

**FIGURE 5:**
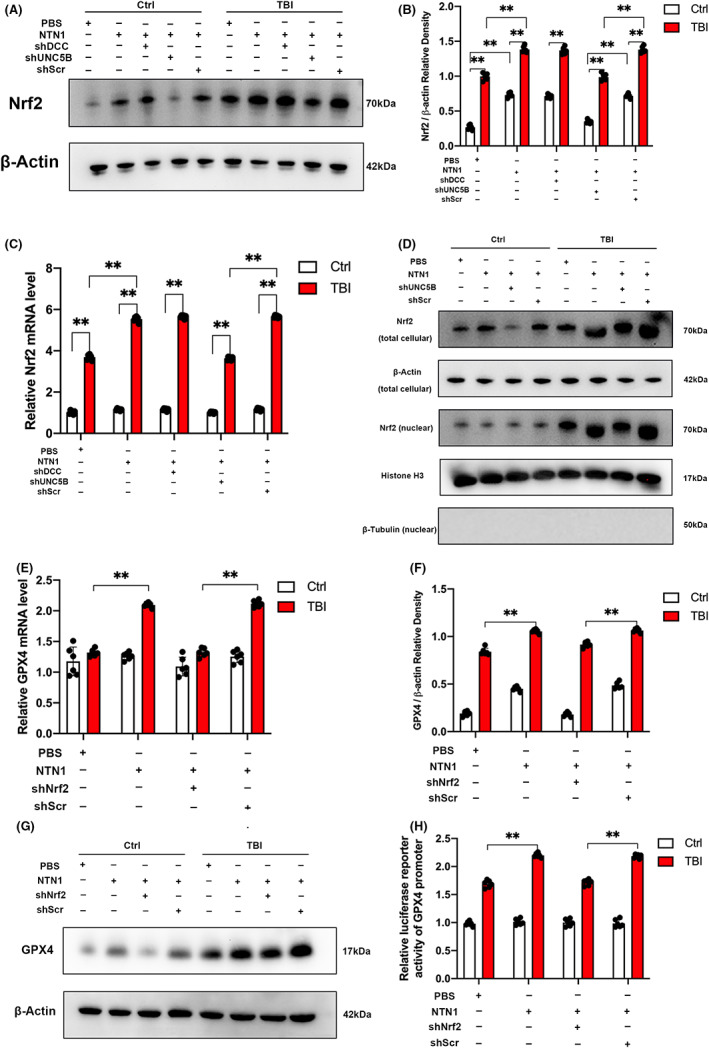
NTN1 upregulated Nrf2 and promoted the Nrf2 nuclear translocation via UNC5B receptor in cell stretch model. Nrf2 enhanced GPX4 transcription in cell stretch model. (A, B) Nrf2 protein content determined by western blot in SH‐SY5Y cell line. β‐Actin was used as control. Data were analyzed using the Mann–Whitney U test. **p < 0.01. (*n* = 6 each) (C) Nrf2 mRNA content in SH‐SY5Y cell line. Quantitative real‐time PCR was carried out to determine the Nrf2 mRNA content. β‐Actin was used as an internal control and the results were presented as fold change of Ctrl+PBS group. The data for each group conformed to a normal distribution. Data are analyzed using the one‐way ANOVA with LSD post‐hoc test. **p < 0.01. (*n* = 6 each) (D) Nrf2 protein content determined by western blot in total cellular lysates and nuclear lysates. β‐Actin was used as control in total cellular. Histone H3 and β‐Tubulin was used as control in nuclear. (E) GPX4 mRNA content in SH‐SY5Y cell line. Quantitative real‐time PCR was carried out to determine the GPX4 mRNA content. β‐Actin was used as an internal control and the results were presented as fold change of Ctrl+PBS group. The data for each group conformed to a normal distribution. Data are analyzed using the one‐way ANOVA with LSD post‐hoc test. **p < 0.01. (*n* = 6 each) (F, G) GPX4 protein content determined by western blot in SH‐SY5Y cell line. β‐Actin was used as control. The data for each group conformed to a normal distribution. Data are analyzed using the one‐way ANOVA with LSD post‐hoc test. **p < 0.01. (*n* = 6 each) (H) Luciferase reporter activity for promoter of GPX4 gene measured in SH‐SY5Y cell stretch model after Nrf2 shRNA treatment. The results are presented as fold change of Ctrl+PBS group. The data for each group conformed to a normal distribution. Data are analyzed using the one‐way ANOVA with LSD post‐hoc test. **p < 0.01. (*n* = 6 each).

We investigated the signal transduction process with mechanical stretch model. Previous results demonstrated that NTN1 regulates the transcription of GPX4. Therefore, we tried to find transcription factors related to NTN1. It is well‐known that Nrf2 is a transcription factor closely related to lipid peroxidation, and it can upregulate the expression of GPX4. Therefore, we assessed the expression of Nrf2 with qPCR and WB after application of exogenous NTN1. The results showed that NTN1 increased the mRNA expression and protein levels of Nrf2 regardless of mechanical damage (Figure 5A–C).

When stress occurs, Nrf2 uncouples from Keap1, migrates from the cytoplasm to nucleus, and combines with the specific promoter fragment to promote the transcription of target gene. Therefore, we separately measured the Nrf2 levels in whole cells and the nucleus. WB results showed that the level of Nrf2 in the nucleus increased after the application of NTN1 (Figure 5D). Similar findings were further acquired by immunofluorescence staining (Figure S5A).

Next, we investigated the expression of GPX4 in vitro experiments. The results of qPCR (Figure 5E), WB (Figure 5F,G), and immunofluorescence staining (Figure S5B) showed that NTN1 increased the GPX4 mRNA expression and protein level, which was consistent with the results obtained from CCI model in mice. We used the dual‐luciferase reporter system to detect the transcription of GPX4 promoter region, and found that the transcription efficiency of GPX4 promoter region increased after exogenous NTN1 application (Figure 5H). In addition, we used lentivirus‐Nrf2 shRNA to inhibit the expression of Nrf2, and then reassessed the expression of GPX4 with qPCR, WB, IF, and dual‐luciferase reporter system. We found that the effect of exogenous NTN1 was offset (Figures 5E–H, S5B), confirming that NTN1 regulated the expression of GPX4 through Nrf2.

NTN1 has two different types of receptors: DCC receptor and UNC5 receptor family. UNC5B receptor is the most common one in the UNC5 receptor family. Therefore, we mainly investigated the role of DCC receptor and UNC5B receptor in vitro experiments. We transfected cells with lentivirus‐UNC5B shRNA and lentivirus‐DCC shRNA, and then evaluated the expression of Nrf2. We found that UNC5B shRNA can offset the effect of NTN1 on the expression of Nrf2, while DCC shRNA had no such effect (Figures 5A–D, S5A). Therefore, we proved that NTN1 regulated the expression of Nrf2 and GPX4 through the UNC5B receptor.

### 3.6 NTN1 could not ameliorate neuronal ferroptosis when UNC5B/Nrf2 pathway inhibited

**FIGURE 6:**
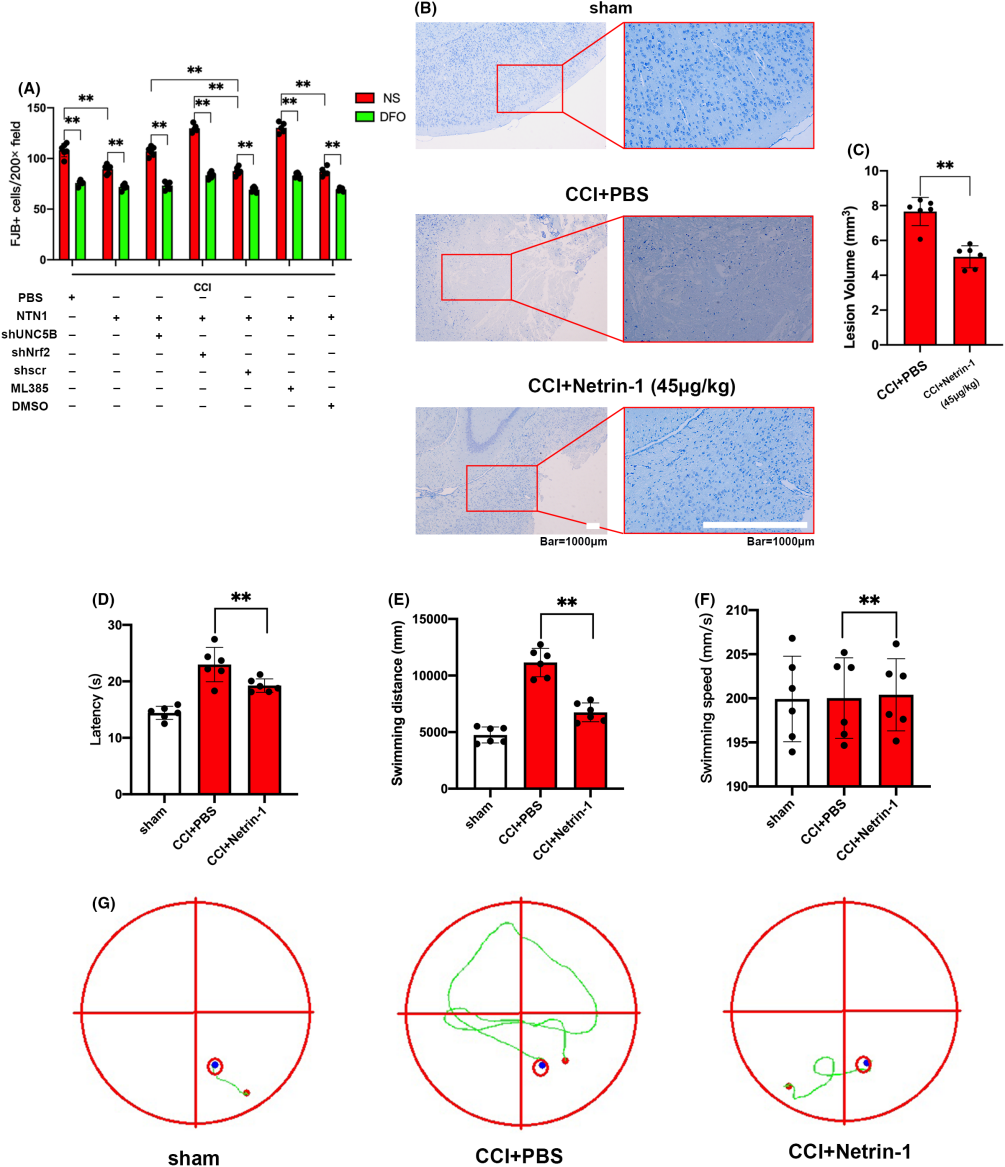
NTN1 could not ameliorate ferroptosis when UNC5B/Nrf2 pathway inhibited. Supplementing NTN1 could decrease the lesion area and improve behavioral performance of mice after CCI. (A) Quantitative analysis of FJB‐positive cells. The data for each group conformed to a normal distribution. *p* value was determined by ANOVA with LSD post‐hoc test. **p < 0.01. There was no difference in body weight between mice in each group. DFO treatment: *n* = 5 (CCI+PBS group and CCI+NTN1+DMSO group, 1 mouse died respectively), *n* = 6 (CCI+NTN1 group, CCI+NTN1+shUNC5B group, CCI+NTN1+shNrf2 group, CCI+NTN1+shScr group and CCI+NTN1+ML385 group). NS treatment: *n* = 5 (CCI+NTN1+shNrf2 group, CCI+NTN1+ML385 group and CCI+NTN1+DMSO group, 1 mouse died respectively), *n* = 6 (CCI+PBS group, CCI+NTN1 group, CCI+NTN1+shUNC5B group and CCI+NTN1+shScr group). (B, C) Representative images of Nissl stained brain sections. Scale bar is 1000 μm. Histograms show the quantitative analyses of lesion volume. There was no difference in body weight between mice in each group. The data for each group conformed to a normal distribution. Data are analyzed using Student's *t* test. **p < 0.01 (*n* = 6 each). (D–G) Representative swimming tracks of the Morris water maze test 4 weeks after CCI. Histograms show the quantitative analyses of latency, swimming distance and swimming speed. There was no difference in body weight between mice in each group. The data for each group conformed to a normal distribution. Data are analyzed using ANOVA with LSD post hoc test. **p < 0.01 (*n* = 6 each).

In order to explore the role of UNC5B/Nrf2 pathway in ferroptosis in CCI model, we treated mice with AAV–UNC5B shRNA, AAV–Nrf2 shRNA (AAV–Scramble shRNA served as control), and Nrf2 specific inhibitor ML385 (DMSO served as control), and reassessed the role of exogenous NTN1. Compared with the control groups, UNC5B shRNA, Nrf2 shRNA, and ML385 all counteracted the effect of exogenous NTN1 in reducing the number of FJB‐positive cells. Interestingly, the effect of UNC5B shRNA, Nrf2 shRNA or ML385 could be counteracted by DFO application (Figure 6A, S6A). Meanwhile, UNC5B shRNA, Nrf2 shRNA, and ML385 counteracted the effect of exogenous NTN1 on reducing MDA (Figure S6B) and ROS (Figure S6C) levels and increasing GSH (Figure S6D) levels in CCI model.

### 3.7 Supplementing NTN1 decreased the lesion area and improved behavioral performance of mice after CCI

We evaluated the lesion area of mice using Nissl staining 3 weeks after CCI and found that NTN1 reduced the loss of neurons (Figure 6B,C). Behavioral experiments were also conducted after CCI. The results of balance beam test showed that mice with exogenous NTN1 application demonstrated fewer foot slips and shorter walking time compared with control group (Figure S6E,F). The results of the water maze showed that mice supplemented with NTN1 had shorter latency and swimming distance compared with control group, but there was no significant difference in swimming speed (Figure 6D–G). These results indicated that NTN1 can improve the prognosis of mice after CCI.

## 4 DISCUSSION

Now, a large number of studies investigated the complex role of Netrin‐1 in the regulation of neuroprotection. Here, we demonstrated that Netrin‐1 had the neuroprotective effect after TBI and inhibited ferroptosis via activating the UNC5B/Nrf2 pathway. (Graphical abstract).

Nuclear Nrf2 is a transcription factor associated with oxidative stress. As an evolutionarily conserved intracellular defense mechanism, Nrf2 participates in carcinogenesis and cellular protection by regulating cellular metabolism and inflammation.^20^ The role of Nrf2 was observed in many neurological disorders. In SAH models, Nrf2 was observed to be upregulated and expected to be a target of several antioxidant factors. Conversely, Nrf2 overexpression was detected in glioma^21^ and this might be associated with the tumor proliferation.^22^ Similar conclusions were reached in TBI models. The dysregulation of Nrf2 pathway was demonstrated to be related to the inflammatory response.^23^ Our experiments in mouse CCI model established that inhibition of Nrf2 by AAV–shNrf2 administration suppressed the anti‐lipid peroxidation effects of Netrin‐1. This suggested that Nrf2 was downstream targets of Netrin‐1 to exert anti‐oxidative effects.

Based on previously reported studies, Nrf2 played an important role in the regulation of ferroptosis. As a protective factor against oxidative stress, Nrf2 alleviated ferroptosis by mitigating lipid peroxidation.^24^ Meanwhile, Nrf2 increased intracellular cystine levels by regulating the cystine/glutamate antiporter SLC7A11.^25^ In this way, Nrf2 modulates the activity of GPX4 and inhibits ferroptotic signal transduction. In our study, Nrf2 was observed to be bound to the promoter sequence of GPX4 except for SLC7A11. Furthermore, our study investigated the migration and orientation of Nrf2 in cell stretch injury model and provided direct evidence that Nrf2 participated in the regulation of neuronal ferroptosis after TBI. Using a mouse CCI model, we demonstrated that AAV–shNrf2 administration counteracted the effect of Netrin‐1 to alleviate neuronal ferroptosis. Thus, regulators of Netrin‐1 might represent potential targets in the anti‐ferroptotic treatment of TBI. A previous study reached similar conclusions in a model of spinal cord injury (SCI). Zinc increased the expression of GPX4 by activating Nrf2 after SCI, attenuated lipid peroxidation, and inhibited neuronal ferroptosis. These results suggested that Nrf2 might be a common signal transduction pathway for multiple ferroptosis inhibitors.^26^

Previous studies observed that ferroptosis was involved in the pathogenesis of secondary injury after TBI.^27^ GPX4 is one of the most important ferroptosis regulatory proteins. The imbalance of excitatory amino acids and the production of oxygen radicals after TBI can impair the metabolism of intracellular cystine, thereby adversely affecting GPX4.^28^, ^29^ In the mouse CCI model, we evaluated the expression of GPX4 at different time points and found that GPX4 levels showed upregulation 7 days after injury. This suggested that there may be a complex feedback regulation mechanism surrounding GPX4, and Netrin‐1 may be a key molecule in the regulation network. Expectedly, such a protective effect may be related to the higher GPX4 expression and lipid peroxidation inhibition.

Netrin‐1 exerted axon guidance through the differential expression of DCC receptor and UNC5 receptor family. High expression of UNC5B enhanced tumor proliferation and metastasis. This may suggest the effect of UNC5B in preventing cell death. Similar protection was observed in Parkinson's disease^30^ and proved to be related to the regulation of inflammation and oxidative stress in SAH model.^17^ Our further study revealed the role of UNC5B in the regulation of ferroptosis in vitro and in vivo models of TBI, suggesting that neuroprotectant can be developed from UNC5B. It is worth noting that the expression of UNC5B receptor in vascular endothelium can interfere with endothelial function,^31^, ^32^ and its expression in glial cells was related to neuroimmune. Future research is warranted to reveal the complex role of UNC5B in different cells in central nervous system. Interestingly, the UNC5B receptor can induce apoptosis in the absence of Netrin‐1, which may be related to the regulation of p53.^33^ This suggests that there may be a complex regulatory network associated with the cytoprotective effects of Netrin‐1/UNC5B signaling.

In addition to mechanical damage, the upregulation of Netrin‐1 can also be observed in SAH models,^18^ reflecting the potential of the nervous system to repair external damage. In our study, Netrin‐1 was shown to improve motor and cognitive function after CCI in mice. The effect of neuronal protection on behavioral performance after TBI is well known. However, previous studies have proposed that damage and repair of white matter also have an important impact on functional recovery. Interleukin‐4^34^ and ethyl pyruvate^35^ can promote post‐traumatic functional recovery by improving white matter integrity and remodeling. Our study focused on the neuronal protective effect of Netrin‐1, and further studies should evaluate its impact on white matter repair after TBI. In addition, the high expression of Netrin‐1 in CNS tumors suggests its role in tumor immune escape and migration.^36^ Therefore, we believe that Netrin‐1 is a potential neuroprotective factor. However, we have not observed the expression of Netrin‐1 in clinical cases, nor have we sought a suitable drug carrier, so there are some limitations in our study. Previous studies proved the inhibitory effect of Netrin‐1 on apoptosis and autophagy^37^ and our research supplemented it. It is believed that the prevention of ferroptosis based on Netrin‐1 is a feasible scheme for treatment of TBI. In sum, we discovered a molecular mechanism and therapeutic target that provide clues for the application of ferroptosis in the treatment of TBI.

## 5 CONCLUSIONS

The present study showed that Netrin‐1 was upregulated in mice CCI model and had neuroprotective effects at early stages. Furthermore, we discovered the new function of Netrin‐1 receptor UNC5B in the oxidative stress signaling pathway. Mechanistically, we established that Netrin‐1 can inhibit ferroptosis and improve neurological functions through the regulation of oxidative stress after TBI. We further revealed that the protective strategy of Netrin‐1 could be offset via downregulation or inhibition of GPX4. Specifically, we discovered that Netrin‐1 promoted the transcription of GPX4 by facilitating Nrf2 nuclear translocation.

## CONFLICT OF INTEREST

The authors declare no competing financial interests exist.

## Supporting information


Appendix S1

## Data Availability

The data that support the findings of this study are available from the corresponding author upon reasonable request.
