# Supplementary material for: Netrin‐1 upregulates GPX4 and prevents ferroptosis after traumatic brain injury via the UNC5B/Nrf2 signaling pathway
Source: CNS Neurosci Ther. 2022 Dec 5;29(1):216–27. doi: 10.1111/cns.13997 (PMC9804081; doi:10.1111/cns.13997)
Supplement: Supplementary file 1 — Appendix S1 [file CNS-29-216-s001.pdf]

Supporting Information

Supplementary Methodology

Materials and Animals

Exogenous NTN1 (1109-N1-025 and 6419-N1-025, R&D Systems, USA), Desferrioxamine (DFO) (Novartis, Switzerland), RSL3 (HY-100218A, MedChemExpress, USA), ML385 (SML1833, Sigma-Aldrich, USA) were used in this study.

The animals were obtained from Shanghai Jie-Si-Jie Laboratory Animal Co., Ltd. and were approved by the Animal Care and Experiment Committee of the School of Medicine, Shanghai Jiao Tong University. Male C57BL/6 mice (6-8 weeks old, 20-25 g) were randomized and coded. Mice were maintained on a temperature 22-25°C, humidity 50%, 12-hour light/dark cycle and specific-pathogens-free (SPF) conditions for at least 7 days prior to surgery. All animals were allowed food and water ad libitum.

Drug administration

Netrin-1 was added to PBS buffer to produce a final concentration of 100 μg/ml and then intravenously injected into mice 1-hour after CCI. Control groups received equal volumes of PBS at the corresponding time points. After the cell mechanical stretch modeling, cells were cultured in serum-free medium containing 100 μg/ml Netrin-1 for 24 hours. The medium free of Netrin-1 was added to control groups.

We started DFO treatment on mice 7 days before CCI. DFO was added to saline at a concentration of 500 mg/ml and intravenously injected into mice every 12 hours at doses of 50 mg/kg until sacrifice. The vehicle-treated group received the same volume of saline solution.

RSL3 was added to DMSO at a concentration of 5 mg/ml and ML385 was added to DMSO at a concentration of 10 mg/ml. RSL3 and ML385 was intravenously injected into mice 1 hour after CCI at doses of 5 mg/kg and 30 mg/kg respectively. DMSO served as control.

shRNA treatment and intracerebroventricular injection

In vivo gene knockdown was achieved by Adeno-associated virus 9 (AAV9) based short hairpin RNA (shRNA) and in vitro gene knockdown was achieved by lentivirus-based shRNA. In brief, we constructed the recombinant plasmid by inserting cDNA between the inverted terminal repeats of AAV9 or lentivirus genome. Helper plasmids were co-transfected with recombinant plasmid into 293T cells to package the vector. The vector was harvested at 48 h post-transfection and was purified by virus purification kit (Cell Biolabs, USA). Virus titer was determined using the Rapid Titer Kit (Clontech, USA). 1×10^{10} copies of AAV9-Vector were injected into the lateral ventricle 14 d before CCI and lentivirus was added to the media at a MOI of 100 72 h before modeling. The target sequences were presented in **Supplementary Table 1**. The knockdown efficiency was validated by WB.

Intracerebroventricular (ICV) injection was performed as previously described[20] with some modifications. Mice were anesthetized and placed in a stereotaxic apparatus. The stereotactic coordinates were as follows: anteroposterior (AP), −0.4 mm; mediolateral (ML), −1.0 mm; dorsoventral (DV), −3.0 mm from the bregma for injection into the left lateral ventricle. Animals were injected with 10 μl using a syringe with a 0.52 mm needle. Virus was injected over 10 min, and the needle was left in place for 10 min prior to withdrawal. Two weeks were needed for successful transfection before CCI.

RNA-sequencing

The samples were obtained from the injured cortex 3 days after CCI and processed for RNA sequencing. Specifically, cortex were collected after saline perfusion and washed in PBS. The tissue was placed into pre-chilled RNase-free tubes and frozen at −80°C. Following sequencing procedures were performed by Majorbio Co. Ltd (Shanghai, China).

Slice preparation

The mice were anesthetized and then transcardially perfused with saline (4°C) and 4% paraformaldehyde (4°C) sequentially. The skin and skull were stripped, and the whole brain was harvested, fixing in 4% paraformaldehyde (4°C) for 6 hours. After that, the brains were dehydrated sequentially in 20% and 30% sucrose solution (4°C) for 24 hours. Finally,

the brain tissues were cut into 20 μm-thick serial coronal sections and stored at -80°C.

Transmission electron microscopy (TEM)

TEM was used to examine the alterations in mitochondrial morphology of the injured neurons. The mice were anesthetized and then transcardially perfused with saline. The brain tissues were quickly removed and placed in the electron microscope fixing solution (Simuwu, China) for 4 hours. After 3 times rinsing with 0.1 M phosphate buffer (PB), fixing in 1% osmium acid/0.1 M PB (PH7.4) for 2 h (20°C), 3 times rinsing with 0.1 M PB, the tissues were sequentially dehydrated with 50%, 70%, 80%, 90%, 95%, 100%, 100% alcohol and 2 times 100% acetone. The tissue samples were then oriented and placed in a 60 °C oven for 48 h. The ultrathin sections (60-80 nm) were cut with an ultramicrotome and stained by 2% uranium acetate saturated alcohol solution and lead citrate for 15 minutes. Sections were dried at room temperature and examined under a transmission electron microscope.

Measurement of malondialdehyde (MDA), reactive oxygen species (ROS), and glutathione (GSH) levels

MDA assay kit (S0131, Beyotime, China) was used to measure the MDA content in mouse brain tissue. The principle of this kit is the color reaction of MDA and thiobarbituric acid to give a red product, having a maximum absorbance at 535nm. The ipsilateral cortex tissues were sufficiently broken and lysed on ice for 30 minutes, and then centrifuged at 12000g for 10 minutes to take the supernatant. BCA kit was used to detect the total protein concentration. According to the kit instructions, the absorbance was measured at 500nm and the MDA content was expressed as nmol/mg prot.

GSH assay kit (S0053, Beyotime, China) was used to measure the GSH content. The principle of this kit is that GSH can react with the chromogenic substrate DTNB to produce yellow TNB and GSSG. Briefly, we collected the ipsilateral cortex tissues, measured the absorbance at 412nm and calculated the GSH content by standard curve according to the instructions.

ROS assay kit (E004-1-1, Jiancheng, China) was used to measure the level of ROS.

The principle of the kit is that DCFH-DA probe can be oxidized by ROS into the fluorescent substance DCF. The absorbance was measured at 500nm and the ROS content was expressed as U/mg prot.

Measurement of Netrin-1 levels in cerebrospinal fluid (CSF) of mice

The cerebrospinal fluid of mice was taken to determine the content of Netrin-1. The mice were sacrificed by CO2 inhalation and the foramen magnum was exposed. We punctured a hole at the base of the skull and obtained the CSF. Only the clear liquid was collected for further use. The CSF was then harvested by centrifugation. Netrin-1 assay kit (SEB827Mu, Cloud-Clone, China) was used to evaluate the Netrin-1 content. Briefly, Netrin-1 antibody was precoated onto 96-well microplates to prepare a solid-phase carrier and the determination of Netrin-1 concentrations was performed by ELISA test. We measured the OD value at 450nm wavelength and calculated the Netrin-1 content by standard curve according to the instructions.

Western-blot and nuclear-cytoplasm separation

Western-blotting was used to assess specific protein levels. For mice, the ipsilateral cortex tissues were sufficiently broken and lysed in RIPA buffer and PMSF with sonication. Samples were lysed for 30 min on ice and centrifuged at 12500 g for 30 min at 4°C to collect the supernatant. For the cells, RIPA buffer and PMSF were added to each culture dish after PBS rinsing. Cells were harvested using a scraper 5 min later, collected into a tube, lysed for 30 min on ice and centrifuged at 12000 g for 10 min at 4°C to collect the supernatant.

After collection, BCA assay kit (Beyotime, China) was used to detect the protein concentrations. The extracted proteins were separated by electrophoresis with the 10% SDS-PAGE and transferred into a polyvinylidene difluoride (PVDF) membrane in a wet electron transfer device. The membrane was blocked at room temperature for 1 h with 5% non-fat milk, incubated with primary antibody overnight at 4°C, washed with TBST three times and then incubated with secondary antibody for 1 h at room temperature. Protein signals were visualized using ECL luminescent detection system. Band density was

quantified by Image J software and data were normalized to β-Actin. The following primary antibodies were used: monoclonal rabbit anti-Netrin-1 (ab126729, 1:1000, Abcam, UK), monoclonal rabbit anti-GPX4 (ab125066, 1:1000, Abcam, UK), monoclonal rabbit anti-FACL4 (ab155282, 1:10000, Abcam, UK), monoclonal rabbit anti-LOX (ab174316, 1:1000, Abcam, UK), monoclonal rabbit anti-Nrf2 (12721, 1:1000, Cell Signaling Technology, USA), monoclonal rabbit anti-DCC (ab273570, 1:1000, Abcam, UK), monoclonal rabbit anti-UNC5B (13851, 1:1000, Cell Signaling Technology, USA), monoclonal rabbit anti-βActin (HRP conjugate) (1:1000, Cell Signaling Technology, USA).

Proteins from different subcellular fractions were prepared by a nucleoplasmic separation kit (P0027, Beyotime, China). Briefly, the cell membrane ruptured due to low osmotic pressure conditions in this method, releasing the cytoplasmic proteins. Nuclei were precipitated by centrifugation and western-blotting was used to evaluate the protein levels.

Quantitative real-time PCR (qRT-PCR)

qRT-PCR was used to quantify the mRNA levels of NTN1, GPX4, and Nrf2. Briefly, the ipsilateral cortex tissues or the SH-SY5Y cells were collected and total RNA was extracted using Trizol reagent (Invitrogen, USA). cDNA was synthesized using 1 μg of total RNA with PrimeScript Reverse Transcriptase (Takara, Japan). The qPCR reaction was performed based on the primers (Sangon, China) and the RR420A kit (Takara, Japan). qPCR was performed using the following thermo-cycling procedure: 95˚C for 5 sec, 60˚C for 30 sec, repeated for 40 cycles. Each sample was detected 3 times and the mean value was obtained. β-Actin was used as an internal control. The primers are presented in **Supplementary Table 2**.

Dual-luciferase reporter gene assay

Dual-luciferase reporter gene assay was used to determine the GPX4 promoter activity. The GPX4 promoter sequence was amplified from pcDNA3.1-GPX4 plasmid and cloned into pGL3 luciferase reporter vectors (Youbio, China). The SH-SY5Y cells were co-transfected with the constructed pGL3 and pRL-TK vector (Youbio, China) for 24h and lysed according to the instructions of Dual Luciferase Reporter Assay Kit (Vazyme, China).

Absorbance of the opaque 96-well plate was read at a wavelength of 350-700 nm using SpectraMax M2 spectrophotometer (Molecular Devices, USA). Three replicate holes were set for each sample and the pRL-TK vector was used as an internal control. The values were normalized to the Ctrl group.

Immunofluorescence staining

Immunofluorescence staining was used to visualize the protein localization. The brain sections or the petri dish containing the adherent cells were fixed with 4% paraformaldehyde for 15 min at room temperature, washed with TBST 3 times, permeabilized with 0.2% Triton X-100/TBST for 20 min, washed with TBST 3 times again, blocked in 10% donkey serum for 1 hour at room temperature and then incubated with primary antibody overnight at 4°C. The samples were protected from light after 3 times washing and incubated with secondary antibody at room temperature for 1 h. The slides were sealed with anti-fluorescence quenching sealing tablets containing DAPI and then observed under fluorescence microscope. The following primary antibodies were used: polyclonal chicken anti-Netrin-1 (ab39370, 1:100, Abcam, UK), monoclonal rabbit anti-GPX4 (ab125066, 1:200, Abcam, UK), monoclonal rabbit anti-Nrf2 (12721, 1:200, Cell Signaling Technology, USA).

Nissl staining

Nissl staining was used to evaluate the damaged area 3 weeks after CCI modeling. The sections were washed with distilled water twice and stained with Nissl staining solution (C0117, Beyotime, China) for 30 min at room temperature. After washing with distilled water twice, dehydrating with 95% ethanol twice, and transparentizing with xylene twice, the sections were sealed with neutral gum. We evaluated the lesion area by Image J software. Specifically, we added the lesion area of each slice and multiplied it by the slice thickness to estimate the lesion volume.

Morris water maze

Four weeks after CCI modeling, the learning and memory functions were evaluated

using the Morris water maze test. The maze was a circular pool with a diameter of 120 cm and a depth of 50 cm. There was a white platform with a diameter of 6 cm and a height of 30 cm on the southwest quadrant of the pool. The pool was filled with water and titanium dioxide was added to the water to make the water white. The platform was immersed 1 cm below the water surface. In each trial, a mouse was positioned in one of the four directions (east, south, west, and north) facing the wall and then released to swim. There were two criteria for terminating the trial: the mouse found the platform and rested on the platform for 10 seconds, or the mouse did not find the platform within 60 seconds. Each mouse was trained for 5 consecutive days, four times per day, with 4-minute interval between trials. On the sixth day, the camera was placed above the maze to record the movement of each mouse, and the track analysis software was used to calculate the results, including swimming distance, latency and swimming speed.

Beam-walking test

Beam-walking test was used to evaluate the motor function of mice 7 days after CCI modeling. The beam was a square wooden stick with a length of 1 m and a width of 14 mm, whose one side was placed in a black square box. In each trial, a mouse was placed on the other side of the beam so that it entered the square box. Each mouse was trained for two consecutive days, three times per day, with 2-hour interval between trials. On the third day, the walking time and the number of slips for the mouse to enter the box through the beam in 60 s were recorded. Mice that failed to enter the box or the walking time exceed 60 s were both recorded as 60 s.

Supplementary Table 1 Target sequences of each shRNA

Gene name	Target sequence (5’-3’)
NTN1	CATGGAGCTCTACAAGCTT
GPX4	GTGGATGAAGATCCAACCC
NRF2	GCCCAUUGAUGUUUCUGAUTT
UNC5B	CCGGCCACACAGATCTACTTCAATTCAAGA
	GATTGAAGTAGATCTGTGTGGTTTTTTG

Supplementary Table 2 Primers used for qRT-PCR analysis

Gene Name	Sequence (5'-3')
mNTN1	Forward: GCCTTCCTCACCGACCTCAATAAC
	Reverse: CTTCTTGCCGAGCGACAGAGTG
mGPX4	Forward: GCCTGGATAAGTACAGGGGTT
	Reverse: CATGCAGATCGACTAGCTGAG
hGPX4	Forward: CGGAATTCATGAGCCTCGGCCGCCTTTG
	Reverse: CCGCTCGAGGAAATAGTGGGGCAGGTCCT
hNRF2	Forward: GCCCAUUGAUGUUUCUGAUTT
	Reverse: AUCAGAAACAUCAAUGGGCTT
mβ-Actin	Forward: GGCTGTATTCCCCTCCATCG
	Reverse: CCAGTTGGTAACAATGCCATGT
hβ-Actin	Forward: AGATGTGGATCAGCAAGC
	Reverse: TCATCTTGTTTTCTGCGC

Supplementary Figure Legends

Supplementary Figure 1

NTN1 expression was upregulated after CCI.

(a) Experimental design in mouse CCI model. (b) Immunofluorescence assessment of NTN1 expression in mice injured cortex. NTN1 is labeled green and DAPI (nucleus marker) is labeled blue. Scale bar is 400 μm.

Supplementary Figure 2

Ferroptosis of injured cortex after CCI.

(a) Transcriptome sequencing and KEGG analysis of CCI+PBS group and CCI+Netrin-1 group (n=6 each). (b) Representative images of Fluoro-Jade B (FJB) stained brain sections at specific time points after CCI. Scale bar is 1000 μm. (c-e) MDA, ROS and GSH content in the injured cortex at different time points after CCI. The data for each group conformed to a normal distribution. Data were analyzed using the one-way analysis of variance (ANOVA) with Least Significant Difference (LSD) post-hoc test. **$P < 0.01$. There was no difference in body weight between mice in each group. n=5 (12h, 3d and 3w after CCI, 1 mouse died respectively), n=6 (sham group, 3h, 1d and 7d after CCI).

Supplementary Figure 3

NTN1 ameliorated ferroptosis in the injured cortex after CCI.

(a) Representative images of Fluoro-Jade B (FJB) stained brain sections. Scale bar is 1000 μm. (b-d) MDA, ROS and GSH levels in the mice injuried cortex. The data for each group conformed to a normal distribution. Data were analyzed using the one-way analysis of variance (ANOVA) with Least Significant Difference (LSD) post-hoc test. $^{**}P < 0.01$. There was no difference in body weight between mice in each group. n=5 (CCI group and CCI+shNTN1 group, 1 mouse died respectively), n=6 (sham group and CCI+shScr group). (e) Representative images of Fluoro-Jade B (FJB) stained brain sections. Scale bar is 1000 μm. (f-h) MDA, ROS and GSH levels in the mice injuried cortex. The data for each group conformed to a normal distribution. Data were analyzed using the one-way analysis of variance (ANOVA) with Least Significant Difference (LSD) post-hoc test. $^{**}P < 0.01$. There was no difference in body weight between mice in each group. n=5 (CCI+5 μg/kg NTN1 group and CCI+45 μg/kg NTN1 group, 1 mouse died respectively), n=6 (other groups).

Supplementary Figure 4

NTN1 ameliorated cortical ferroptosis by upregulating GPX4.

(a-b) Histogram shows the quantitative analysis of Acsl4 and LOX protein content determined by western blot. The data for each group conformed to a normal distribution. P value was determined by ANOVA with LSD post-hoc test. $^{**}P < 0.01$. There was no difference in body weight between mice in each group. n=5 (CCI group and CCI+shNTN1 group, 1 mouse died respectively), n=6 (sham group and CCI+shScr group). (c-d) Histogram shows the quantitative analysis of Acsl4 and LOX protein content determined by western blot. The data for each group conformed to a normal distribution. P value was determined by ANOVA with LSD post-hoc test. $^{**}P < 0.01$. There was no difference in body weight between mice in each group. Sham group: n=6 each. CCI group: n=5 (CCI+15 μg/kg NTN1 group, 1 mouse died), n=6 (CCI+PBS group, CCI+5 μg/kg NTN1 group and CCI+45 μg/kg NTN1 group). (e-g) MDA, ROS and GSH levels in the mice injuried cortex. Data were analyzed using the Mann-Whitney U test. $^{**}P < 0.01$. There was no difference

in body weight between mice in each group. n=5 (CCI+NTN1 group, CCI+NTN1+shGPX4 group and CCI+NTN1+RSL3 group, 1 mouse died respectively), n=6 (other groups).

Supplementary Figure 5

NTN1 upregulated Nrf2 and promoted the Nrf2 nuclear translocation via UNC5B receptor in cell stretch model. Nrf2 enhanced GPX4 transcription in cell stretch model.

(a) Immunofluorescence assessment of Nrf2 expression in SH-SY5Y cell line. Nrf2 is labeled red and DAPI (nucleus marker) is labeled blue. Scale bar is 200 μm. (b) Immunofluorescence assessment of GPX4 expression in SH-SY5Y cell line. GPX4 is labeled green and DAPI (nucleus marker) is labeled blue. Scale bar is 200 μm.

Supplementary Figure 6

NTN1 could not ameliorate ferroptosis when UNC5B/Nrf2 pathway inhibited. Supplementing NTN1 could decrease the lesion area and improve behavioral performance of mice after CCI.

(a) Representative images of Fluoro-Jade B (FJB) stained brain sections. Scale bar is 1000 μm. (b-d) MDA, ROS and GSH levels in the mice injuried cortex. The data for each group conformed to a normal distribution. Data were analyzed using the one-way analysis of variance (ANOVA) with Least Significant Difference (LSD) post-hoc test. **P < 0.01. There was no difference in body weight between mice in each group. n=5 (CCI+PBS group, CCI+NTN1+shUNC5B group and CCI+NTN1+shScr group, 1 mouse died respectively), n=6 (other groups). (e-f) Histograms show the quantitative analyses of foot slips and time in the beam walking test. Data were analyzed using the Mann-Whitney U test. **P < 0.01. There was no difference in body weight between mice in each group. n=5 (CCI+Netrin-1 group, 1 mouse died), n=6 (other groups).
